# Rhizosphere and Straw Return Interactively Shape Rhizosphere Bacterial Community Composition and Nitrogen Cycling in Paddy Soil

**DOI:** 10.3389/fmicb.2022.945927

**Published:** 2022-07-07

**Authors:** Ya-Hui Zhao, Ning Wang, Meng-Kang Yu, Jian-Guang Yu, Li-Hong Xue

**Affiliations:** ^1^Key Laboratory of Agro-Environment in Downstream of Yangtze Plain, Ministry of Agriculture, P.R. China, Jiangsu Academy of Agricultural Sciences, Nanjing, China; ^2^School of the Environment and Safety Engineering, Jiangsu University, Zhenjiang, China; ^3^Jiangsu Key Laboratory for Food Quality and Safety, State Key Laboratory Cultivation Base of Ministry of Science and Technology, Nanjing, China; ^4^Henan Institute of Science and Technology, Xinxiang, China

**Keywords:** rhizosphere, straw return, bacterial communities, nitrogen cycling, interactively

## Abstract

Currently, how rice roots interact with straw return in structuring rhizosphere communities and nitrogen (N) cycling functions is relatively unexplored. In this study, paddy soil was amended with wheat straw at 1 and 2% w/w and used for rice growth. The effects of the rhizosphere, straw, and their interaction on soil bacterial community composition and N-cycling gene abundances were assessed at the rice maturity stage. For the soil without straw addition, rice growth, i.e., the rhizosphere effect, significantly altered the bacterial community composition and abundances of N-cycling genes, such as archaeal and bacterial *amoA* (*AOA* and *AOB*), *nirK*, and *nosZ*. The comparison of bulk soils between control and straw treatments showed a shift in bacterial community composition and decreased abundance of *AOA, AOB, nirS*, and *nosZ*, which were attributed to sole straw effects. The comparison of rhizosphere soils between control and straw treatments showed an increase in the *nifH* gene and a decrease in the *nirK* gene, which were attributed to the interaction of straw and the rhizosphere. The number of differentially abundant genera in bulk soils between control and straw treatments was 13–23, similar to the number of 16–22 genera in rhizosphere soil between control and straw treatment. However, the number of genera affected by the rhizosphere effect was much lower in soil amended with straw (3–4) than in soil without straw addition (9). Results suggest possibly more pronounced impacts of straw amendments in shaping soil bacterial community composition.

## HIGHLIGHTS

- Bulk and rhizosphere differed in bacterial communities and N-cycling genes.
- Straw amendment altered bacterial community composition and N-cycling.
- High numbers of genera were affected by straw in the rhizosphere than in the bulk.
- Fewer genera were affected by the rhizosphere when straw was returned to the soil.
- Straw amendments affected bacterial communities more pronouncedly than the rhizosphere.

## Introduction

The rhizosphere is a hotspot of root–microbe interaction that critically links with soil functions and plant nutrient acquisition (Moreau et al., 2019). *Via* root exudation, rhizodeposition, and nutrient uptake, plant roots are powerful drivers of microbial community assembly (Hu et al., 2018). Previous studies have shown that rhizosphere microbial communities are distinct from bulk soil (Li et al., 2018). Rhizosphere effects on microbial communities could be a result of taxonomic shifts, which in turn shape patterns of ecological interactions regulating the structure, function, and potential resilience of soil microbial communities (Wu et al., 2017; Langarica-Fuentes et al., 2018; Yuan et al., 2018). In particular, the potential for adaptive plant–microbe feedback is relevant for plant nitrogen (N) acquisition, which is a limiting nutrient in most agricultural ecosystems (Pérez-Izquierdo et al., 2019). Rhizosphere microbes can increase soil N supply to the plant and have substantial feedback on plant productivity *via* regulating N-cycling processes (Emmett et al., 2019; Pérez-Izquierdo et al., 2019). Recent studies have shown that the rhizosphere is enriched in functional genes related to N fixation (*nifH*), nitrification (*amoA, hao*), and denitrification (*narG, nirS*/*nirK, norB*, and *nosZ*) compared to bulk soils (Ai et al., 2013; Li et al., 2014; Wang et al., 2017). In agricultural systems, in-depth knowledge on factors controlling rhizosphere microbial communities and N-cycling may help inform agricultural management systems that harness rhizosphere processes to recouple plant–microbial N supply and demand to limit N losses (Ollivier et al., 2011; Bowles et al., 2015; Yu et al., 2015) while guaranteeing the health and productivity of plants (Manoeli et al., 2017; Zhalnina et al., 2018; Ding et al., 2019; Emmett et al., 2019; Maarastawi et al., 2019).

In agricultural systems, soil microbial communities and nutrient cycling are usually affected by the interactions of agricultural managements (e.g., straw return) and the rhizosphere environment (Schmidt et al., 2019). Among various agricultural managements, straw return is widely applied to increase soil organic carbon and soil fertility (Meng et al., 2017; Wang et al., 2018, 2020). Many studies have monitored changes in the soil microbial community following straw application (Zhao et al., 2016; Chen et al., 2017; Yang et al., 2019). In the early stage after straw return, bacteria dominate the crop residue decomposition, while fungi dominate at the later stage (Paterson et al., 2008; Marschner et al., 2011). In particular, crop residue return in flooded paddy soil significantly increases the relative abundance of Firmicute and decreases the abundance of Proteobacteria, thereby altering microbial community compositions (Wang et al., 2018). In addition, *via* altering the microorganism, straw return increases N fixation and reduces N losses, thereby increasing plant-N supply (Choudhury and Kennedy, 2004; Maeda et al., 2010; Chen et al., 2017). The responses of the microbial communities in bulk soils to agricultural management could help predict biogeochemical processes at the field or ecosystem scale (McGuire and Treseder, 2010; Graham et al., 2016). However, rhizosphere microbial communities, which are of critical importance for agricultural productivity, are shaped by interactions between agricultural management and plant selection processes (Schmidt et al., 2019). So far, how rice roots interact with straw return in structuring rhizosphere communities and N-cycling functions is relatively unexplored. The difference in microbial communities and N-cycling between bulk and rhizosphere soils without straw return can be contributed to rhizosphere effects, while the shifts of bulk soil induced by straw addition can be regarded as straw effects. In comparison, the interaction effects of rhizosphere and straw can be reflected by the variation in microbial communities and N-cycling in the rhizosphere with the straw return.

To explore the rhizosphere, straw, and their interaction effects on paddy soil microbial communities and N-cycling, a representative paddy soil was collected, added with wheat straw at two application rates (1 and 2%), and then used for rice growth under greenhouse conditions. Soil microbial communities and N-cycling-related gene abundances were measured for both bulk and rhizosphere soils at the rice maturity stage. The specific objectives were to (1) determine bacterial community diversity, composition, and N-cycling genes in bulk and rhizosphere with and without straw addition and (2) assess the relative roles of the rhizosphere, straw, and their interaction in influencing paddy soil bacterial diversity, composition, and N-cycling genes. This knowledge can contribute to better managing rhizosphere interactions and promoting both plant productivity and agroecosystem sustainability.

## Materials and Methods

### Soil and Straw

Rice soil was collected from paddy fields of Taizhou, Jiangsu, China. The soil was derived from the fluvial deposit (Wang et al., 2021). After collection, the soil was air-dried, sieved (2 mm mesh), and homogenized for soil property analyses and rice growth. Soil properties were determined, including pH (6.79, 1:5 of soil-to-water ratio), total organic carbon (TOC, 23.7 g kg^–1^), total nitrogen (TN, 1.15 g kg^–1^), alkali-hydro nitrogen (AN, 36.7 mg kg^–1^), available phosphorus (AP, 27.4 mg kg^–1^), and available potassium (AK, 260 mg kg^–1^) (Wang et al., 2021). Wheat straw was collected from a wheat production field during the harvest season. After air-drying, the straw was crushed to powder form and analyzed for contents of carbon (C, 46.5%) and N (0.48%). The ratio of C/N in the straw was 96.9.

### Experiment Setup

To assess the influences of straw return on soil microbial community, three soil treatments were performed in triplicates, namely, (1) control treatment without straw amendment, (2) straw treatment by adding 1% w/w wheat straw (dry weight basis, dw), and (3) straw treatment by adding 2% w/w wheat straw (dw). Straw was added at 1 and 2% to represent the straw return of ∼12 and 24 t ha^–1^ under field conditions.

To allow for rice cultivation in a greenhouse, soils without or with straw addition were filled into pots using rhizo-bags separating bulk and rhizosphere. Initially, soils without or with straw amendment were added with fertilizers, including urea (250 mg N kg^–1^ dry soil), calcium superphosphate (60 mg P kg^–1^ dry soil), and potassium chloride (100 mg K kg^–1^ dry soil). Then, 1.5 kg dry weight (dw) of soil was transferred into rhizo-bags (30 μm nylon mesh, diameter 7.5 cm × height 23 cm), which were then placed in the center of polyvinyl chloride pots (diameter 15 cm × height 23 cm). Then, another 4.5 kg dw of soil was weighed into the pot to fill the space outside of the rhizo-bags. The rhizo-bags allow small molecular substrates to penetrate but prohibit roots to penetrate, thereby being a good way to divide rhizosphere from bulk soils (Nie et al., 2015). Soils were flooded for 3 days prior to the transplantation of rice seedlings (cv. Nanjing 9108). Uniform seedlings 3 days after germination were transplanted, with one seedling in each pot. Following transplantation, rice was daily flooded to maintain ∼2 cm overlying water during the period from seedling to flowering, while at the filling stage, rice was under the alternation of wetting and drying conditions to improve rice yield. At the maturity stage, i.e., 122 days after transplantation, rice was moved out from the rhizo-bags. The rhizosphere soils inside the rhizo-bags were collected and sieved (2 mm) to remove roots. The bulk soils outside the rhizo-bags were also collected and sieved. The rhizosphere and bulk soils were used for the analyses of soil properties, soil bacterial community structure, and N-related functional genes.

### Soil Property Analyses

The dried soils were mixed with CO_2_-removed water at a soil:water ratio of 1:5 (w/v) prior to measurements of pH and electrical conductivity (EC) using corresponding electrodes. The soil was oxidized with potassium dichromate and measured for TOC using titration with ferrous ammonium sulfate. Soil TN was measured by an elemental analyzer (Vario MAX CNS, Elementar, Germany). AN was measured using the alkali solution diffusion method (Lu, 2000). Concentrations of soil ammonium (NH_4_^+^) and nitrate (NO_3_^–^) were analyzed using ion chromatography (ICS-3000, Dionex, United States) following extraction with 2 M KCl. AK and AP were measured using an inductively coupled plasma optical emission spectrometer (Optima 5300DV, PerkinElmer, United States).

### Bacterial Community Analyses

A total of 18 soil samples, collected from bulk and rhizosphere of control and straw treatments, were subjected to soil bacterial community diversity and composition analysis using Illumina sequencing. Genomic DNA in the soil samples was extracted using a FastDNA SPIN Kit. The bacterial 16S rRNA gene was amplified using primers [515F (5′–GTGCCAGCMGCCGCGG–3′), 907R (5′–CCGTCAATTCMTTTRAGTTT–3′)] that target V4–V5 region. The thermal profile of PCR included an initial denaturation at 95°C for 3 min, 30-cycle denaturing at 95°C for 30, annealing at 58°C for 1 min, extension at 72°C for 45 s, and a final extension step at 72°C for 10 min. Equal amounts of PCR products from different samples (barcoded) were mixed, purified, and quantified prior to Illumina sequencing at Shanghai Lingen Genomics Institute, China (Wang et al., 2015).

The sequence data were processed using the Quantitative Insights into Microbial Ecology toolkit (QIIME, version 1.7.0) the data presented in the study are deposited in the GenBank Data repository, accession number PRJNA847362. After removing the low quality or ambiguous reads, the identified sequences were then clustered into operational taxonomic units (OTUs) at a 97% similarity level, and the representative sequence (i.e., the most abundant sequence) was assigned to taxonomy by an RDP classifier (version 2.2; Wang et al., 2007). Based on the OTU matrix, principal coordinate analysis (PCoA) was performed to assess the differences in overall community composition between bulk and rhizosphere with different amounts of the straw amendment (Lozupone et al., 2005). Moreover, heatmaps based on genus level and redundancy analysis (RDA) were conducted using R software (version 2.14.0) and the community ecology package vegan (2.0–4) to identify soil properties (including pH, EC, TOC, TN, AN, NH_4_^+^–N, NO_3_^–^–N, AP, and AK) contributing to the altered bacterial community (Oksanen et al., 2013). Envfit function (999 permutations) analyses were used to remove environmental variables that insignificantly contributed to the total soil bacterial community variance. Significant differences in the major genus of bacterial community composition between rhizosphere and bulk soils or between control and straw treatments were assessed by Student’s *t*-test (*p* < 0.05).

### N-Cycling Functional Genes

Primer pairs of *nifHF/nifHR* (Rosch et al., 2002), Arch-*amoA*-F/Arch-*amoA*-R (Francis et al., 2005), *amoA-1F/amoA-1R* (Rotthauwe et al., 1997), *nirK-F1aCu/nirK-R3Cu* (Throbäck et al., 2004), *nirS-cd3aF/nirS-R3cd* (Hallin and Lindgren, 1999), and *nosZ*-F*/nosZ-1622*R (Throbäck et al., 2004) were used to quantify *nifH*, archaeal and bacterial *amoA* (*AOA* and *AOB*), *nirK, nirS*, and *nosZ* genes in soil samples using real-time PCR system (ABI 7500, American), respectively (Supplementary Table 2). Following DNA extraction, each gene was amplified using a 20 μl system, i.e., 10 μl SYBR^®^ Premix Ex Taq™ II (2×), 0.4 μl ROX reference Dye II (50×), 0.8 μl forward primer (10 μM), 0.8 μl reverse primer (10 μM), 6 μl ddH_2_O, and 2 μl template DNA (20 ng). Real-time PCR conditions for target genes are listed in Supplementary Table 2. Gene standard curves were constructed based on gradient dilutions of standard plasmids containing each gene with known copy numbers. To ensure correct amplification, DNA extracts were highly diluted to eliminate the inhibition. To ensure no contamination during qPCR, negative controls without DNA templates were included in each patch of gene amplification. In addition, gene quantification of each sample was performed in three parallel real-time PCR reactions, with reactions of efficiencies > 90% and correlation coefficients *r*^2^ > 0.99 being accepted. Target gene copy numbers of each sample were calculated from the standard curves and expressed on a basis of soil dw (copies g^–1^ dw soil).

## Results

### Soil Properties

For control treatment without straw return, there were significant differences in soil properties between rhizosphere and bulk (Table 1), indicating the sole rhizosphere effects. Soil TN (1.04 g kg^–1^) and NO_3_^–^–N (21.6 mg kg^–1^) concentrations were significantly higher in the rhizosphere than in bulk soil (0.98 g kg^–1^ and 9.65 mg kg^–1^). In contrast, the rhizosphere showed significantly lower NH_4_^+^–N concentration (6.18 mg kg^–1^) than bulk soil (7.91 mg kg^–1^). The pH of the rhizosphere was 7.09, significantly higher than that of bulk soil (6.97). There were insignificant differences in other soil properties.

**TABLE 1:** Chemical characteristics of the rhizosphere and bulk soils without and with straw addition at the rice maturity stage (*n* = 3).

Treatment	pH	EC μm cm^−1^	TOC (g kg^–1^)	TN (g kg^–1^)	AN (mg kg^–1^)	NH_4_^+^-N (mg kg^–1^)	NO_3_^–^ N (mg kg^–1^)	AP (mg kg^–1^)	AK (mg kg^–1^)
BS0	6.97 ± 0.05*^c^*	435 ± 3.30*^a^*	8.85 ± 0.56*^d^*	0.98 ± 0.02*^e^*	88.6 ± 9.77*^a^*	7.91 ± 0.26*^b^*	9.65 ± 0.33*^e^*	27.9 ± 3.45*^a^*	70.0 ± 2.65*^cd^*
BS1	7.10 ± 0.07*^ab^*	353 ± 51.4*^a^*	9.93 ± 0.11*^c^*	1.01 ± 0.03*^de^*	108 ± 5.27*^a^*	7.63 ± 0.40*^b^*	11.8 ± 0.43*^d^*	31.3 ± 2.62*^a^*	115 ± 11.5*^b^*
BS2	7.11 ± 0.06*^ab^*	398 ± 78.1*^a^*	10.5 ± 0.05*^b^*	1.14 ± 0.01*^b^*	114 ± 5.01*^a^*	10.0 ± 0.85*^a^*	14.5 ± 0.24*^c^*	29.5 ± 0.74*^a^*	146 ± 21.7*^a^*
RS0	7.09 ± 0.05*^b^*	390 ± 9.59*^a^*	8.43 ± 0.32*^d^*	1.04 ± 0.02*^cd^*	104 ± 10.6*^a^*	6.18 ± 0.05*^c^*	21.6 ± 1.22*^b^*	28.4 ± 1.09*^a^*	47.3 ± 20.6*^d^*
RS1	7.18 ± 0.07*^ab^*	372 ± 55.3*^a^*	10.0 ± 0.18b*^c^*	1.05 ± 0.01*^c^*	98.8 ± 2.78*^a^*	6.22 ± 0.45*^c^*	23.1 ± 1.69*^b^*	30.1 ± 4.40*^a^*	79.0 ± 16.0*^c^*
RS2	7.22 ± 0.08*^a^*	412 ± 20.9*^a^*	11.1 ± 0.12*^a^*	1.23 ± 0.01*^a^*	95.5 ± 27.4*^a^*	6.31 ± 0.63*^c^*	34.4 ± 0.33*^a^*	27.1 ± 2.39*^a^*	140 ± 12.1*^ab^*

*BS0, bulk soil without straw addition; BS1, bulk soil with addition of 1% straw addition; BS2, bulk soil with addition of 2% straw addition; RC, rhizosphere soil without straw addition; RS1, rhizosphere soil with addition of 1% straw addition; RS2, rhizosphere soil with addition of 2% straw addition; EC, electrical conductivity; TOC, total organic carbon; TN, total nitrogen; AN, available nitrogen; NH_4_^+^, ammonium; NO_3_^–^, nitrate; AP, available phosphorus; AK, available potassium. Different superscript letters indicate significant (p < 0.05) differences in various treatments.*

For bulk soil, changes in soil properties with straw addition were observed (Table 1), indicating the sole straw effects. Excluding EC, AN, and AP, significant increases were observed in bulk soil pH (from 6.97 to 7.11), TOC (from 8.85 to 10.5 g kg^–1^), TN (from 0.98 to 1.14 g kg^–1^), NH_4_^+^–N (from 7.91 to 10.0 mg kg^–1^), NO_3_^–^–N (from 9.65 to 14.5 mg kg^–1^), and AK (from 70.0 to 146 mg kg^–1^) with straw addition.

Changes in rhizosphere soil properties were also observed following straw addition (Table 1), indicating the interactions of rhizosphere and straw. Like bulk soil, EC, AN, and AP insignificantly changed with straw addition. In addition, NH_4_^+^–N concentrations (6.22–6.31 mg kg^–1^) of straw-treaded rhizosphere soils were also not significantly different from that of control rhizosphere soil (6.18 mg kg^–1^). In comparison, straw addition significantly increased rhizosphere soil pH (from 7.09 to 7.22), TOC (from 8.43 to 11.1 g kg^–1^), TN (from 1.04 to 1.23 g kg^–1^), NO_3_^–^–N (from 21.6 to 34.4 mg kg^–1^), and AK (from 47.3 to 140 mg kg^–1^).

Nitrogen content in the root, shoot, and rice from paddy soils with different amounts of straw addition are listed in Supplementary Table 1, showing that straw addition significantly increased the N content in the root, shoot, and rice from paddy soils.

### Bacterial Community Diversity and Composition

**FIGURE 1:**
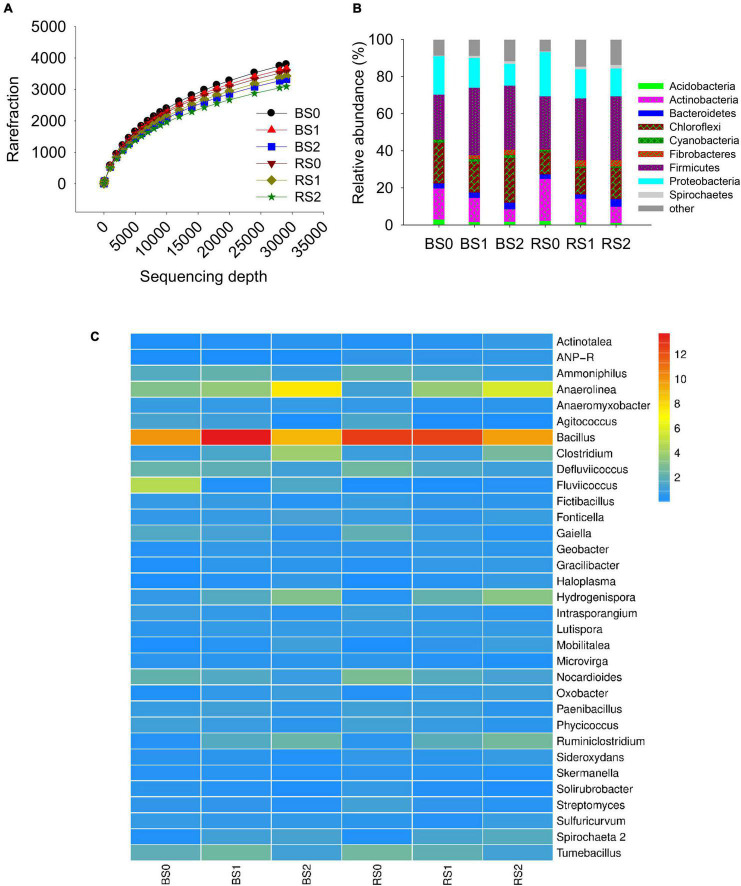
Rarefaction distribution **(A)**, and the relative abundances in class **(B)** and genera **(C)** level of bacterial community composition in bulk and rhizosphere of paddy soils with and without straw amendment. Each column represents the mean value of triplicate analyses. BS0, bulk soil without straw addition; BS1, bulk soil with addition of 1% straw addition; BS2, bulk soil with addition of 2% straw addition; RS0, rhizosphere soil without straw addition; RS1, rhizosphere soil with addition of 1% straw addition; RS2, rhizosphere soil with addition of 2% straw addition.

Rarefaction of observed species showed that even at a sequencing depth of 290,704, the diversity of soil bacteria continued increasing rapidly with increasing sequencing depth (Figure 1A), suggesting the high diversity of soil bacteria. Insignificant differences in Chao, Shannon, Simpson, and Coverage index at the sequencing depth were observed between bulk and rhizosphere soils or between control and straw treatments (Table 2), suggesting that rhizosphere, straw, and their interactions had no effects on internal bacterial community diversity.

**TABLE 2:** Diversity and abundance indices of bacterial community with and without straw addition.

Treatments	Richness index	Diversity index		Coverage index
	**Chao**	**Shannon**	**Simpson**	**Coverage**
BS0	5458 ± 219*^a^*	6.893 ± 0.162*^a^*	0.00458 ± 0.00222*^a^*	0.9584 ± 0.0047*^a^*
BS1	5427 ± 285*^a^*	6.879 ± 0.063*^a^*	0.00392 ± 0.00040*^a^*	0.9634 ± 0.0093*^a^*
BS2	5165 ± 323*^ab^*	6.696 ± 0.124*^ab^*	0.00478 ± 0.00056*^a^*	0.9699 ± 0.0047*^a^*
RS0	5110 ± 217*^ab^*	6.827 ± 0.143*^ab^*	0.00409 ± 0.00121*^a^*	0.9620 ± 0.0060*^a^*
RS1	5446 ± 338*^a^*	6.696 ± 0.047*^ab^*	0.00469 ± 0.00040*^a^*	0.9702 ± 0.0051*^a^*
RS2	4740 ± 236*^b^*	6.613 ± 0.091*^b^*	0.00477 ± 0.00141*^a^*	0.9692 ± 0.0065*^a^*

*BS0, bulk soil without straw addition; BS1, bulk soil with addition of 1% straw addition; BS2, bulk soil with addition of 2% straw addition; RS0, rhizosphere soil without straw addition; RS1, rhizosphere soil with addition of 1% straw addition; RS2, rhizosphere soil with addition of 2% straw addition. Different superscript letters indicated significant (p < 0.05) differences in various treatments.*

The major bacterial community compositions at class level were Actinobacteria (3.53–11.1%), Alphaproteobacteria (3.84–11.1%), Anaerolineae (6.22–22.0%), Bacilli (13.4–22.7%), Clostridia (6.99–20.4%), Deltaproteobacteria (3.21–5.19%), and Gammaproteobacteria (4.19–10.7%) (Figure 1B). The major bacterial community compositions at genus level were *Anaerolinea* (1.12–7.57%), *Bacillus* (9.67–13.7%), *Clostridium* (0.71–3.97%), *Defluviicoccus* (1.04–2.54%), *Fonticella* (0.58–1.20%), *Nocardioides* (0.87–2.84%), *Tumebacillus* (1.16–2.60%), with *Bacillus* being the dominant genera (Figure 1C).

**FIGURE 2:**
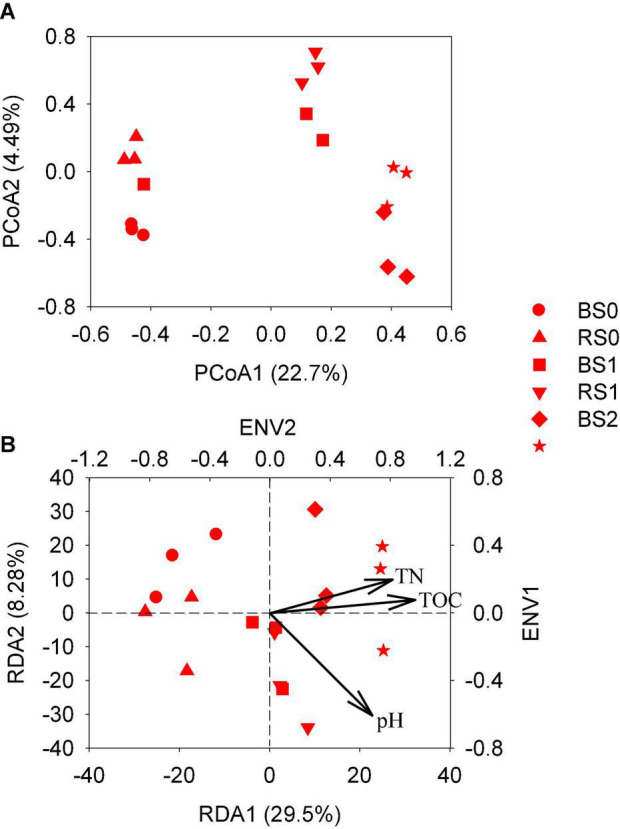
Principal coordinate analysis (PCoA) and redundancy analysis (RDA) of bacterial community composition **(A,B)** in rhizosphere and bulk from paddy soils with and without straw amendment. BS0, bulk soil without straw addition; BS1, bulk soil with addition of 1% straw addition; BS2, bulk soil with addition of 2% straw addition; RS0, rhizosphere soil without straw addition; RS1, rhizosphere soil with addition of 1% straw addition; RS2, rhizosphere soil with addition of 2% straw addition.

The PCoA showed that the bacterial community compositions of the rhizosphere soils were significantly different from that of the bulk soils for control treatment or straw treatment at 1% (Figure 2A). However, for straw addition at 2%, the bacterial community compositions of bulk soils were overlapped with those of rhizosphere according to Adonis analysis (*p* > 0.05).

Regardless of bulk and rhizosphere soils, the bacterial community compositions of soils with straw addition were significantly separated from those of soils without straw addition (*p* < 0.05). This indicated that straw addition significantly changed the bacterial community composition of both bulk and rhizosphere soils, while root growth significantly altered the bacterial community composition for soils without and with a lower rate of straw addition. At higher straw application, the relatively stronger influence of straw might have obscured the effect of the rhizosphere on bacterial community compositions.

By envfit function (999 permutations), soil pH, TN, and TOC were found to be significantly correlated with the bacterial community composition at the OTU level (Figure 2B). RDA showed that variation in these three factors together explained 41.5% of soil bacterial community composition variation in bulk and rhizosphere soils with or without straw addition.

### Number of Differentially Abundant Genera

**FIGURE 3:**
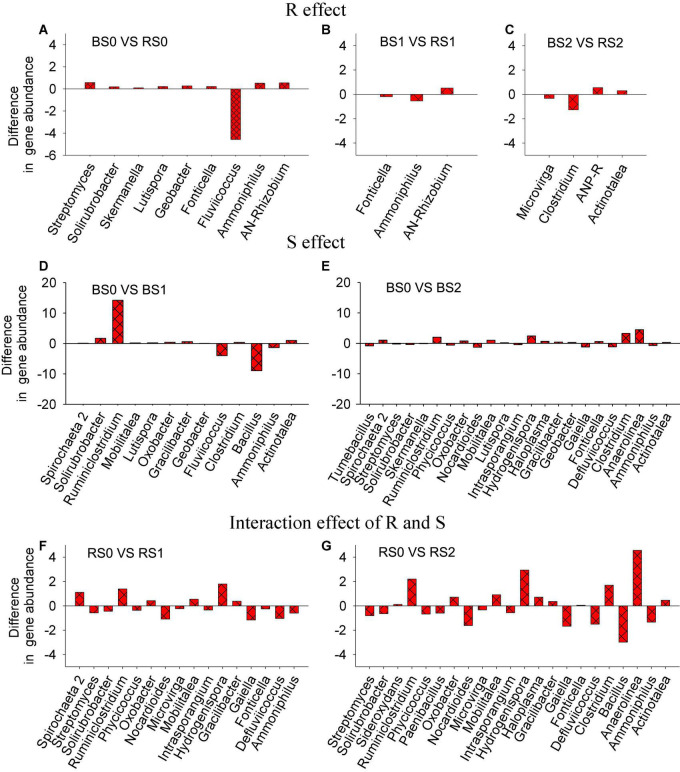
Responses of genera abundances in bacterial community composition to rhizosphere (R, **A–C**), straw (S, **D,E**) effects, or their interaction (R × S, **F,G**). R effect can be reflected by the difference in microbial communities between bulk and rhizosphere soils without straw return; S effect can be reflected by the variation in microbial communities in bulk soil with and without straw return; the interaction effect of R and S can be reflected by the variation in microbial communities in rhizosphere with straw return.

The genera whose relative abundance significantly varied with straw, rhizosphere, or their interaction effects were identified (Figure 3). By comparing bulk and rhizosphere soils without straw addition, a total of nine genera showed significant (*p* < 0.05) differences in relative abundances. However, by comparing bulk and rhizosphere soils treated with 1 or 2% straw, three or four genera were identified (Figures 3A–C), suggesting that the rhizosphere effect might be weakened when straw was returned to the soil.

For the straw effect, the number of differentially abundant taxa between bulk soils without and with the addition of straw at 1% was 13, while the number was 23 when straw was added at 2% (Figures 3D,E). These results suggested that compared to the rhizosphere effect, the straw effect affected more genera, highlighting the possible stronger impacts of straw amendments on soil bacterial community composition.

For the interaction effect of rhizosphere and straw, we observed 16 and 22 differentially abundant genera in rhizosphere soils when comparing control and 1% straw treatments and comparing control and 2% straw treatments (Figures 3F,G).

### N-Cycling Functional Genes

**FIGURE 4:**
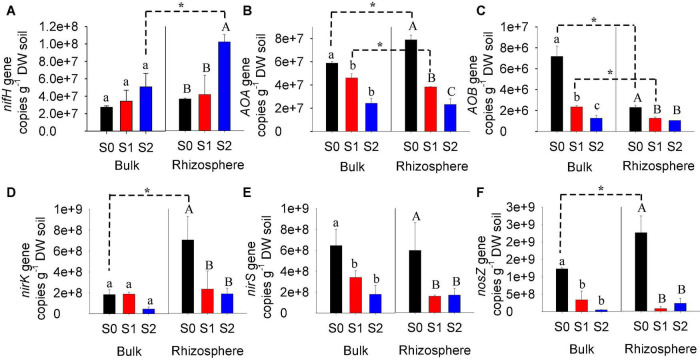
Genes copy number of N-cycling genes (*nifH, AOA, AOB, nirK, nirS*, and *nosZ*) in bulk and rhizosphere of paddy soils with and without straw amendment **(A–F)**. Different upper letters indicated significant (*p* < 0.05) differences in various treatments; *Indicates significant (*p* < 0.05) differences between bulk and rhizosphere. S0, soil without straw addition; S1, soil with 1% straw addition; S2, soil with 2% straw addition.

Bulk and rhizosphere soils without or with straw amendment varied in N-cycling functional genes (Figure 4). For control soils without straw addition, the abundances of archaeal *amoA, nirK*, and *nosZ* genes were significantly higher in the rhizosphere, but the abundance of bacterial *amoA* gene was lower compared to bulk soils. There were insignificant differences in the abundances of *nifH* and *nirS* genes between bulk and rhizosphere soils without straw addition. In comparison, for soils with 1% straw application, the gene abundances of archaeal *amoA* and bacterial *amoA* were lower in the rhizosphere than in bulk, while other genes were similar between rhizosphere and bulk. For soils with 2% straw application, *nifH* gene abundance was significantly higher in the rhizosphere compared to bulk soils.

For bulk soils, straw amendment significantly decreased the gene abundances of archaeal *amoA* and bacterial *amoA*, which decreased at higher extents at a higher straw application rate (Figure 4). The gene abundances of *nirS* and *nosZ* were also significantly decreased in bulk soils with straw addition, but unaffected by the straw application rate. No obvious difference in *nifH* and *nirK* gene abundances was observed in bulk soils with the straw amendment.

Like bulk soils, the straw amendment also significantly decreased the gene abundance of archaeal *amoA*, bacterial *amoA, nirK, nirS*, and *nosZ* in rhizosphere soils (Figure 4). At a higher rate of straw addition, the gene abundance of archaeal *amoA* was decreased to a higher extent, while the gene abundance of bacterial *amoA, nirK, nirS*, and *nosZ* was decreased at similar extents when 1 and 2% straw were amended. In comparison, the gene abundance of *nifH* was increased by 2% straw addition, while it was not affected by 1% straw addition.

## Discussion

In this study, we assessed how root and straw return acted individually or in combination in shaping bacterial community composition and N-cycling functions. Rhizosphere is a unique zone and plays a vital role in N fixation, nitrification, and denitrification (Arth et al., 1998; Nie et al., 2014; Moreau et al., 2019). We observed that the rhizosphere significantly altered soil bacterial community composition (Figure 2A), and the changes in soil pH, TOC, and TN concentrations were critical factors in shifting the soil microbial community, explaining 41.5% of the total variation of soil bacterial community composition in paddy soil (Table 1 and Figure 2B). Soil characteristics were reported to be important factors in shaping microbial community composition (GraAff et al., 2010; Zhalnina et al., 2015; Ji et al., 2021). The rhizosphere effect could result in the change of soil characteristics (Table 1), thereby obviously altering the microbial community.

With regard to N-cycling functions, the release of oxygen from rice root is favorable for soil nitrification (Armstrong, 1971). Previous studies showed that ammonia-oxidizing archaea were more abundant in the rhizosphere than in bulk soil (Chen et al., 2008), suggesting that *AOA* is more dominant in the rhizosphere paddy soil and more influenced by root exudation (e.g., oxygen, carbon dioxide) than *AOB*. We observed similarly that the rhizosphere was enriched in the *AOA* gene in this study (Figures 4B,C). In addition, root exudates could be used as C sources for denitrifying bacteria growth, thereby increasing the denitrification activity (Baudoin et al., 2003). In this study, we observed that function genes (*nirK* and *nosZ*) related to denitrification were enriched in the rhizosphere (Figures 4D–F), which might depend on the nutrient concentration and habitat selection.

Compared to the rhizosphere effect, the straw return may play a more pronounced role in shaping the bacterial community composition (Figure 3). During the whole stage of rice growth, the degradation of straw could supply C for bacterial growth (Murase et al., 2006). However, at the mature stage, root exudation was relatively low, decreasing the contribution of C from the rhizosphere. Thus, higher numbers of genera were affected by straw return than the rhizosphere effect (Figure 3). Moreover, the shift of bacterial community composition in bulk soils was observed to be stronger at 2% straw than 1% application (Figure 2A), suggesting that the straw effect on the bacterial community composition was more remarkable at a higher amendment rate. This might be related to greater changes in soil pH, TOC, and TN concentration at a higher rate of straw application (Table 1 and Figure 2B). For N-cycling functions, different from the rhizosphere effect, the straw effect decreased the abundance of *AOA, AOB, nirS*, and *nosZ* (Figure 4) since straw addition decreased the available N (Wang et al., 2018).

Under straw amendment, the rhizosphere also significantly altered soil bacterial community composition (Figure 2A). However, higher numbers of genera were affected by the effect of the rhizosphere when the soil was not amended with straw return compared to that with the straw return, suggesting that the rhizosphere effect might be weakened when straw was returned to the soil, possibly due to the more pronounced impacts of straw amendments. In addition, there was no obvious difference in *nifH* between bulk and rhizosphere soils for control treatment, but rhizosphere soil displayed significantly higher *nifH* abundance than bulk when the soil was amended with straw addition (Figure 4A). Straw addition into soils increased soil C/N ratio but decreased soil-available N (Table 1), which might stimulate the N-fixing microorganism to transfer more N into the soil. However, the limit-available N could also decrease the activity of nitrification and denitrification microbes (Xiao and Tang, 2014), thus *AOA* and *AOB* abundance were decreased and no obvious increase in denitrification gene was observed for rhizosphere with straw addition in this study.

A higher rate of straw application shaped the bacterial communities of rhizosphere soils more remarkably. The shift of bacterial community composition and the changed genera in rhizosphere soils were observed to be higher at 2% straw than 1% application (Figures 1B,C, 2A). This might be related to greater changes in pH, TOC, and TN concentration of rhizosphere soils at a higher rate of straw application (Table 1 and Figure 2B). For N-cycling genes, straw addition in the rhizosphere soils decreased the abundance of *AOA, AOB, nirS*, and *nosZ* (Figure 4). However, the response of *nifH* and *nirK* genes to the effect of straw addition in rhizosphere soil was different from their response to straw effect in bulk soil (Figure 4). Compared to bulk soil, the rhizosphere supplies more N to plants, which might stimulate the N-fixing microorganism and further decrease *nirK* gene abundance (Hou et al., 2018; Wen et al., 2019). Thus, the interaction effect of rhizosphere and straw increased the *nifH* gene but decreased the *nirK* gene.

## Conclusion

The rhizosphere interacts with the straw return to shape rhizosphere microbial community composition and N-cycling processes. Compared to the rhizosphere effect, the straw return may play a more pronounced role in shaping the bacterial community composition of rice paddy soil at the mature stage. Reframing research priorities to better understand the rhizosphere and agricultural management interactions has important implications for assessing the ecology and functions of rhizosphere microbial communities, which could help guide plant-oriented strategies to improve productivity and agroecosystem sustainability.

## Data Availability Statement

The data presented in the study are deposited in the GenBank Data repository, accession number PRJNA847362.

## Author Contributions

Y-HZ conducted the incubation experiments, measured soil property and N-cycling functional genes, data analysis, and wrote the manuscript. NW managed the whole project, designed all the experiments, and jointly wrote the manuscript. M-KY measured soil property. J-GY and L-HX helped with manuscript revision. All authors contributed to the article and approved the submitted version.

## Conflict of Interest

The authors declare that the research was conducted in the absence of any commercial or financial relationships that could be construed as a potential conflict of interest.

## Publisher’s Note

All claims expressed in this article are solely those of the authors and do not necessarily represent those of their affiliated organizations, or those of the publisher, the editors and the reviewers. Any product that may be evaluated in this article, or claim that may be made by its manufacturer, is not guaranteed or endorsed by the publisher.
