# Supplementary material for: Rhizosphere and Straw Return Interactively Shape Rhizosphere Bacterial Community Composition and Nitrogen Cycling in Paddy Soil
Source: Front Microbiol. 2022 Jul 7;13:945927. doi: 10.3389/fmicb.2022.945927 (PMC9301285; doi:10.3389/fmicb.2022.945927)
Supplement: Supplementary file 1 [file Table_1.pdf]

Table 1. Nitrogen content in root, shoot and rice from paddy soils with different amounts of straw addition

Plant	Treatment	N（g/kg)
Root	S0	6.11 ±0.3[b]
	S1	6.71 ±0.44[ab]
	S2	7.29 ±0.31[a]
Shoot	S0	8.44 ±0.43[b]
	S1	9.96 ±0.94[a]
	S2	10.5 ±0.82[a]
Rice	S0	11.2 ±0.64[c]
	S1	13.9 ±0.27[b]
	S2	15.9 ±0.40[a]

S0, soil without straw addition; S1, soil with addition of 1% straw addition; S2, soil with addition of 2% straw addition.

Different upper letters indicated significant ($p < 0.05$) differences in root, shoot and rice with different amounts of straw addition.
